# Moral Elevation and Economic Games: The Moderating Role of Personality

**DOI:** 10.3389/fpsyg.2019.01381

**Published:** 2019-06-14

**Authors:** Rico Pohling, Rhett Diessner, Shawnee Stacy, Destiny Woodward, Anja Strobel

**Affiliations:** ^1^Division of Personality Psychology and Assessment, Department of Psychology, Faculty of Behavioural and Social Sciences, Chemnitz University of Technology, Chemnitz, Germany; ^2^Division of Social Sciences, Department of Psychology, Lewis-Clark State College, Lewiston, ID, United States

**Keywords:** need for cognition, engagement with moral beauty, volunteering, dictator game, ultimatum game

## Abstract

Moral elevation is the prototypical emotional response when witnessing virtuous deeds of others. Yet, little is known about the role of individual differences that moderate the susceptibility to experiencing this self-transcendent emotion. The present experiment investigated the role of personality traits as moderators of elevation and its behavioral effects using economic games as a measure for prosocial behavior. One aim was to replicate prior findings on trait Engagement with Moral Beauty as moderator for experimentally induced state elevation. A second aim was to explore new potential moderators that were found to be connected to the moral realm: Honesty-Humility, Agreeableness vs. Anger, and Need for Cognition. Third, the present study is among the few investigating the effects of elevation on different prosocial actions and intentions. A sample of US American college students (*N* = 144) was randomly assigned to either watch a morally uplifting or a humorous video clip. Afterwards, all participants played a dictator game and an ultimatum game, and were asked to volunteer in another time-consuming experiment. In line with our hypotheses, experimentally induced state elevation promoted prosocial behavior, however, only within the dictator game. Also in line with our hypotheses, higher levels of Need for Cognition and higher levels of Engagement with Moral Beauty (but not higher levels of Honesty-Humility and Agreeableness vs. Anger) increased prosocial behavior within the experimental group. In contrast to our hypotheses, none of the investigated personality traits moderated the proneness to experience the state of elevation after seeing an elevating video clip; only the behavioral consequences of elevation were moderated. Our results replicate and extend prior findings on moderators for elevation and exemplify the importance of investigating the role of personality traits in the context of the moral elevation phenomenon.

## Introduction

Putting his own life into great danger, the German industrialist Oskar Schindler saved the lives of 1,200 Jews during the Holocaust by employing them in his factories in Poland and the Protectorate of Bohemia and Moravia (for a detailed protrait, see Crowe, 2004). Schindler spent his entire fortune on bribes and black market purchases to prevent the execution of his workers until the end of World War II. After the end of war, he received support from the Jews he had rescued and was helped to be brought into safety since he was in danger of being arrested as a war criminal due to his membership in the Nazi party and German Abwehr intelligence. Eventually, he even had to declare bankruptcy in 1963 and, from this time on, lived off donations by the so-called Schindlerjuden – the workers and families whose lives he had saved. Schindler died in 1974 and was buried on Mount Zion in Israel, and was the only member of the Nazi party to be honored in this special way. He received several awards for his outstanding example of humanity (cf. Crowe, 2004).

Who is not moved by such a story of courage, self-sacrifice, and loyalty to all humanity? Who does not feel morally uplifted and inspired to do good things when one hears about such acts of moral beauty? The present research aims at contributing to these questions by identifying interindividual differences in the susceptibility to feel moral emotions when witnessing moral excellence, such as the one Oskar Schindler showed. The most prototypical response to such acts of moral beauty is the moral emotion of elevation (Algoe and Haidt, 2009), a positive emotional response that encompasses all the psychological and motivational consequences listed above.

Research on elevation is a young but rapidly growing field; however, a lot of questions still remain unanswered (Pohling and Diessner, 2016; Thomson and Siegel, 2017). In their review article, Pohling and Diessner (2016) concluded that it is insufficiently understood “which personal characteristics increase or decrease the susceptibility to elevation and its behavioral consequences” (p. 421). The present research aims at filling this gap by investigating how *Engagement with Moral Beauty* (EmB; Diessner et al., 2008) and other morality related personality traits, namely *Honesty-Humility* (H-H; Ashton and Lee, 2007), *Agreeableness vs. Anger* (AG; Ashton and Lee, 2007), and *Need for Cognition* (NFC; Cacioppo and Petty, 1982), moderate the susceptibility to moral elevation and its behavioral effects. That is, the current research is among the few studies investigating the moderating role of personality traits not only for elevation itself but also for the prosocial behavior that subsequently is prompted by elevation.

## Moral Elevation

Moral elevation is the emotional response to witnessing virtuous acts of others (Algoe and Haidt, 2009; Diessner et al., 2013). Powerful elicitors of elevation are acts of charity, kindness, love, loyalty, or self-sacrifice (Haidt, 2003b). The central components of elevation that have been theorized and found across studies are the following (cf. Pohling and Diessner, 2016; Thomson and Siegel, 2017): Elevation entails *physical sensations* like a feeling of warmth in the chest, relaxed muscles, and eyes filled with tears (sometimes even a lump in the throat). With regard to *affective reactions*, people feel uplifted, moved, inspired, and report feelings similar to awe and admiration. In terms of *cognition*, elevation broadens the momentary thought-action repertoire by inducing optimistic thoughts about people and humanity. As to short-term *motivational* consequences, elevation induces the tendency to act prosocially and to become a better person, but also to affiliate with others. Thus, elevation is able to prompt prosocial or altruistic^1^ intentions and behavior (Schnall et al., 2010; Schnall and Roper, 2012). However, which kind of prosocial or altruistic behaviors are triggered by elevation has not yet been systematically investigated. Therefore the present research investigates the effects of elevation on three different prosocial variables: prosocial intentions and two forms of cooperation. Finally, as a *long-term consequence* elevation is said to induce upward spirals of positive change (see also Algoe and Haidt, 2009).

A growing body of literature shows that the behavioral outcome of elevation is indeed prosocial or altruistic behavior; this has been measured in a variety of ways, such as cooperative behavior or the tendency to volunteer (e.g., Algoe and Haidt, 2009; Schnall et al., 2010; Aquino et al., 2011). Recent studies exemplified this further by showing that moral elevation boosts prosocial behavior within various economic games (Cova et al., unpublished; Sakai et al., 2016). Several studies could demonstrate that the performance in such games is linked to real-life moral behavior making it a valuable and ecologically valid measure for research (cf. Zhao and Smillie, 2015). Hence, the current study used two well-researched and well-recognized economic games (the dictator and the ultimatum game, for a detailed description of these games, see below) as behavioral measures of cooperative or prosocial behavior to analyze the different effects of elevation and its moderators on such behavior.

Previous experimental studies explored the elevation process in detail (Thomson and Siegel, 2013; Siegel et al., 2014). These studies identified that the experience of elevation as well as its behavioral effects can be moderated by certain aspects of the stimulus material that induces elevation (Thomson and Siegel, 2013; Siegel et al., 2014; Yao and Enright, 2018). If one was exposed to a moral story where the character of the recipient of the moral act was good, it was associated with increased states of elevation and subsequent donation behavior in contrast to stories where the recipient’s character was bad (Thomson and Siegel, 2013). However, the perceived effort of the moral act did not influence the subjective feeling of elevation but did influence its behavioral effects: when participants read about a moral exemplar that needed higher effort to complete a moral act, it caused higher amounts of donation behavior than among those who read about a similar act demonstrating less effort (Thomson and Siegel, 2013). Later research showed that the actual consequences of a particular moral act play also an important role: even if the recipient of a moral act had bad characteristics, actions with good/beneficial consequences increased feelings of moral elevation and subsequent prosocial intentions and behavior (Yao and Enright, 2018).

### Personality as a Moderator of Moral Elevation

Recent narrative reviews underlined the necessity to investigate the antecedents and conditions for experiencing the state of elevation, especially personal characteristics (cf. Pohling and Diessner, 2016; Thomson and Siegel, 2017). However, only little research has been done until now in this regard. Previous studies repeatedly identified that women are more prone to experiencing elevation (cf. Pohling and Diessner, 2016). Further, Diessner et al. (2013) found that the personality trait EmB (Diessner et al., 2008) – which entails frequent and intense appreciation and awe related to the moral excellence of others – moderates the susceptibility to experience moral elevation in response to watching an elevating video-clip. That is, people high on EmB are more prone to experience elevation when witnessing moral beauty.

But not only individual differences in the appreciation of moral beauty can influence levels of the susceptibility to elevation but the centrality of morality for the self appears to also be an important moderating factor. Two experimental studies found that moral identity is a condition of experiencing elevation (Aquino et al., 2011; Lai et al., 2014): chronically or temporally activated schemas of moral identity made people more able to experience the state of elevation or made them recall more acts of moral goodness than people whose moral identity was low (Aquino et al., 2011; Lai et al., 2014). Likewise male patients in treatment for serious antisocial behavior and substance problems showed significantly lower elevation responses when watching an elevating video clip in contrast to a control non-patient group (Sakai et al., 2016) – especially those scoring high on callous unemotional traits operationalized by the “with limited prosocial emotions” specifier of the Diagnostic and Statistical Manual for Mental Disorders-Fifth Edition (DSM-5). Recent studies extended these early findings by identifying narcissism, the instability of self-esteem, and empathy for other’s positive emotions as individual moderators of moral elevation (Thomson, 2017). While the first two attenuated the effects of experimentally induced elevation, the latter fostered them (Thomson, 2017). In sum, these findings exemplify that moral-related personality traits have the power to moderate moral elevation.

The present research validates and goes beyond these prior findings by replicating the moderating role of EmB and by identifying other personality traits that might moderate the susceptibility to experience elevation. In our view, one particular useful template to conceptualize the moral personality is the three-level approach of McAdams (2009). It consists of decontextualized and *broad dispositional traits* (level 1), such as the five factor or the HEXACO model of personality, *characteristic adaptions* (level 2), i.e., “motivational, social-cognitive, and developmental constructs that are more specific and contextualized in time, place and/or social role” (p. 16), and *integrative life narratives* (level 3), which are internalized and evolving life stories that function to provide unity, purpose, and meaning (cf. McAdams, 2009). Consequently, “a moral personality would consist of those traits, adaptions, and stories that best support and sustain a moral life in culture” (McAdams, 2009, p. 23). Furthermore, McAdams (2009) stated that specific profiles of the Big 5 (high Agreeableness and Conscientiousness and at least moderately high Openness to Experience) are associated with high moral functioning. Likewise, specific profiles of the HEXACO personality framework were discussed as expressions of a moral personality (Ashton and Lee, 2007), namely higher levels of H-H and AG.

Ashton and Lee (2007) identified a sixth broad personality factor called H-H which is “the tendency to be fair and genuine in dealing with others, in the sense of cooperating with others even when one might exploit them without suffering retaliation” (p. 156). Complementing the classic five factor model, H-H is able to explain variance in the moral domain over and above the Big 5. For instance, HEXACO outperformed the Big 5 model in predicting workplace delinquency, overt integrity, or sexual harassment (cf. Ashton and Lee, 2007; Ashton et al., 2014). Further, Ashton and Lee (2007) theorized that H-H and AG may represent complementary aspects of reciprocal altruism (see also Zhao and Smillie, 2015) which is particularly interesting for elevation research. Moral elevation was often found to be related to altruistic tendencies (cf. Pohling and Diessner, 2016; Thomson and Siegel, 2017). Therefore, we selected HEXACO H-H and AG as primary candidates to moderate elevation.

HEXACO AG entails the tendency to be forgiving, patient, and tolerant of others and cooperate even if one is exploited by others (for the slight differences in comparison to FFM-Agreeableness see in detail Ashton and Lee, 2007). Therefore, due to its inbuilt prosocial orientation, AG might foster moral elevation and its behavioral effects. On the other hand, high AG was observed to be related to different moral behavior than H-H. While high H-H was related to active-cooperation (directly doing something that helps others, measured with the dictator game, Thielmann and Hilbig, 2018), high AG was related to reactive-cooperation (behavior that reacts to someone’s offensive or non-moral action in a patient and agreeable way without retaliating, measured with the ultimatum game; see in detail, Hilbig et al., 2013). In terms of evolutionary altruism, non-exploitation (active cooperation) and non-retaliation (reactive cooperation) are crucial elements to establish a long-term mutual cooperation with another person. In contrast to the Big 5 model, where prosocial variance is mainly captured by Agreeableness, the HEXACO model differentiates between the aforementioned two forms of prosocial behavior by capturing its variance within two divergent constructs: H-H and AG (Ashton and Lee, 2007; Zhao and Smillie, 2015).

We further posit that the humble, honest, and faithful attitude entailed in HEXACO H-H (Hilbig et al., 2014) can be a beneficial breeding ground for enhancing and fostering moral elevation and its effects. In contrast, people who are low on H-H see themselves as more important than others and seek wealth and status using immoral means (Ashton et al., 2014), which is also reflected by a high negative correlation of H-H and the dark triad traits of Machiavellianism, narcissism, and psychopathy (Lee and Ashton, 2005). Since elevation is a response to moral beauty, low H-H should therefore be a hindrance for perceiving other’s good qualities and, thus, experiencing moral elevation.

In addition to these level 1 constructs according to McAdams (2009) model, we propose that certain characteristic adaptions (level 2, domain-specific traits) will also explain inter-individual differences in the susceptibility for elevation (Haidt, 2003b). One such characteristic adaption is EmB (see above), which can be regarded as an *affective* engagement construct within the moral domain. However, we posit that interindividual differences in *cognitive* engagement should also play an important role on level 2 of the moral personality. One such cognitive engagement construct is NFC (Cacioppo and Petty, 1982). NFC is conceptualized as “stable individual differences in people’s tendency to engage in and enjoy effortful cognitive activity” (Cacioppo et al., 1996, p. 197; see also Cacioppo and Petty, 1982).

There is ample evidence that NFC is a particularly useful predictor of individual differences in information processing and decision-making, with high NFC being, e.g., associated with an enhanced consideration of information quality (for review, see Cacioppo et al., 1996; Petty et al., 2009). With regard to its possible impact on moral behavior, recent evidence suggests that different levels of NFC are associated with individual moral attitudes (McClaren et al., 2009) and predict which situational aspects are considered as morally relevant (Singer et al., 1998). Kinnunen and Windmann (2013) found a positive relation between NFC and displayed moral courage in a group situation. Furthermore, Mussel et al. (2014) observed NFC to be associated with behavioral reactions to unfairness in an ultimatum game with increased acceptance of fair offers and more rejections of unfair ones. Furthermore, NFC was found to be directly related to self-reported moral behavior and to have incremental validity in the prediction of moral behavior over and above moral traits (Strobel et al., 2017). Based on this background one can imagine that carefully considering many perspectives in a morally relevant situation might foster especially the cognitive effects of elevation as individuals high in NFC would more intensely reflect upon the moral acts they are experiencing.

## Aims and Hypotheses

Taken together, research on moral elevation has just begun to identify individual differences in the susceptibility to experience moral elevation. Thus, the present experiment investigates how moral elevation and its behavioral effects are moderated by several personality traits. With respect to this aim, we intend to replicate Diessner et al. (2013); however, the present experiment goes beyond their study since (1) we investigate additional traits which until now have not been explored as moderators of moral elevation and (2) we focus not only on the susceptibility to elevation but also its behavioral effects (i.e., prosocial behavior) in the context of economic games. Furthermore, (3) we systematically investigate the influence of experimentally induced elevation on three different prosocial dependent variables: active cooperation, reactive cooperation, and prosocial intentions.

To measure the behavioral effects of moral elevation we used the framework of economic games. In the so-called dictator game participants have the sole power to split an endowment between themselves and an unknown recipient. In other words, they have the possibility to show *active cooperation*, that is, not exploiting others despite having the power to do so without facing any retaliation (Hilbig et al., 2013). Dictator game giving can therefore legitimately be regarded as an expression of altruism or active prosocial behavior (Baumert et al., 2014; Zhao and Smillie, 2015; Thielmann and Hilbig, 2018). In contrast, in the ultimatum game, the participant takes the role of the responder. A proposer makes a suggestion how to split an endowment between her or himself and the responder. Then, the responder (the participant) has the option to retaliate, when getting unfair offers, by rejecting the offer so that both the proposer and the responder will get nothing. If the responder accepts the offer the split is realized as proposed. Thus, the performance of the participants within the ultimatum game can be regarded as a measure for *reactive cooperation* since accepting relatively low offers represent a form of non-retaliation even in the face of exploitation by others (cf. Hilbig et al., 2013). Thus it represents another but more passive type of prosocial behavior (Zhao and Smillie, 2015). However, while being in the role of the responder within the ultimatum game fairness concerns of the participant seem to play a much greater role than in the dictator game (Baumert et al., 2014). Lastly, as an indicator for *prosocial intentions*, we used the willingness to volunteer as a third dependent variable in our study as has been done in prior elevation research (Cox, 2010; Schnall et al., 2010; Schnall and Roper, 2012). Volunteering is a kind of active prosocial behavior that is voluntary and without direct reward or compensation. However, it is highly socially desirable, which can be seen as a kind of indirect reward. We used this measure as an operationalization for prosocial intentions rather than behavior since we only measured the intention to volunteer. In contrast to the dictator and ultimatum game, it is no performance-based behavioral measure. It is therefore conceptually different from our other measures.

According to the theoretical assumptions and empirical findings described above, we formulate the following hypotheses:

**Hypothesis 1:** Higher states of moral elevation will result in (a) higher active-cooperation, (b) higher reactive-cooperation, and (c) it will foster prosocial intentions.

**Hypothesis 2:** Individuals (a) high on EmB, (b) high on AG, (c) high on H-H, and (d) high on NFC will be more susceptible to experiencing the state of moral elevation in response to watching an elevating video clip than individuals that score lower on these traits.

**Hypothesis 3:** The elevation-induced prosocial intentions and behavior will be more pronounced in individuals who are (a) high on EmB, (b) high on AG, (c) high on H-H, and (d) high on NFC.

## Materials and Methods

### Participants

Of the *N* = 144 participants in this study the experimental group was *n* = 67 and the control group was *n* = 77. The total sample comprised 40% women, 37% men, and 23% participants not indicating gender. The mean age was 20.83 (*SD* = 5.32) ranging from 18 to 50 years. Regarding ethnic background most participants were of Euro descent (61%), and 7% Latino descent, 9% several other ethnic groups including Native American, African or Asian descent, or mixed; 23% participants not indicated any ethnic background. As to beliefs, 52% participants were Christian, 10% Agnostic, 13% reported a variety of beliefs, and 25% indicated no religious beliefs. Participants were diverse in their interests, representing over 20 different majors ranging from Business to the Social Sciences to the Natural Sciences to technical majors. All were students at a small state college in the western United States.

Of the total sample, 142 participants completed the dictator game, 143 completed the ultimatum game, and 141 completed the volunteering question. To reduce experimenter demand artifacts, as Hilbig et al. (2013) recommended, we deleted participants who claimed to allocate all their money in the dictator game or who claimed to accept even a zero offer in the ultimatum game. Accepting zero offers within the UG or donating all money within the DG are outcomes that typically do not occur in bargaining games with monetary incentives (see e.g., Güth et al., 1982; Eckel and Grossman, 1996) so it is likely that these responses represent demand artifacts. Nevertheless, throughout the results section we also report the results found with the untrimmed sample (see below).

After the deletion of these cases and deleting one case with extreme outlier scores on H-H (defined as a *z*-score > 3.29), the final analysis sample consisted of *N* = 118 with *n* = 50 (experimental group) and *n* = 68 (control group). To not further reduce the analysis sample, we did not delete those participants who had missing values on the sociodemographic variables, NFC, EmB, HEXACO, the economic games, or the volunteering question which resulted in slightly different sample sizes for the correlation analysis (see Table 1), the analysis of the main effects of the elevation induction and its behavioral effects (see below), and the moderation analysis (*N* = 85 with *n* = 38 in the experimental and *n* = 47 in the control condition). Throughout the manuscript, we refer to the initial sample (*N* = 144) as *untrimmed sample*, and we will refer to the sample where we deleted the cases described above as *trimmed sample* (*N* = 118).

**Table 1:** Means (standard deviations in parenthesis) and bivariate correlations of all variables.

Variable	1	2	3	4	5	6	7	8	9	10	11.
1. Honesty-Humility	1										
2. Agreeableness (vs. Anger)	0:28*	1									
3. Engagement moral Beauty	0:05	−0:12	1								
4. Need for Cognition	0:08	−0:01	0:18	1							
5. Elevating emotions	0:06	0:06	0:11	0:17	1						
6. Desire to be better	−0:02	0:18†	0:00	0:02	0:69***	1					
7. Elevation factor	0:02	0:15	0:06	0:10	0:90***	0:93***	1				
8. Amusement factor	0:05	−0:04	0:14	0:23*	−0:29***	−0:47***	−0:42***	1			
9. Dictator Game	0:31**	0:22*	0:15	0:15	0:20*	0:19*	0:21*	−0:10	1		
10. Ultimatum Game	−0:05	−0:07	−0:09	−0:01	−0:14	−0:01	−0:06	−0:02	0:02	1	
11. Volunteering	0:05	−0:08	0:22*	0:36***	0:09	−0:06	0:01	0:08	0:12	−0:11	1
Mean	3:50	2:94	5:07	3:27	3:39	3:00	3:20	3:65	42:85	25:28	
(SD)	(.51)	(.54)	(1.22)	(.58)	(1.18)	(1.41)	(1.19)	(1.22)	(19.50)	(21.65)	−

Spearman correlations for dictator/ultimatum game; Pearson correlations for all other continuous scales; Point-biserial correlations for correlations involving volunteering; dictator game: higher values reflect higher active cooperation; ultimatum game: lower values reflect higher reactive cooperation; volunteering: 1 = no, 2 = yes. The correlations depicted are those using the trimmed sample. Due to missing values sample sizes varied for all variables between 89 < N < 117. ***p < 0.001, **p < 0.01, *p < 0.05, †p < 0.10, all tests two-tailed; all ps < 0.05 in bold.

### Procedure

Students in two sections of “Introduction to Psychology” were randomly assigned to the experimental or control conditions. On one day, they filled in the questionnaires measuring EmB, H-H, AG, and NFC; 2 weeks later, they completed the actual experiment. Those in the elevation condition (experimental group) watched a morally uplifting video of a Thai insurance advertisement showing a man helping various people although receiving no direct reward for it, “Heartwarming Thai Commercial” (3:06 min)^2^; those in the amusement condition (control group) watched a humorous video excerpt from the Big Bang Theory television show, “Sheldon Trains Penny” (2:44 min)^3^. Displays of humorous scenes are often used in research to induce mirth or joy (e.g., Schnall et al., 2010), whereas videos or stories displaying acts of charity or kindness or forgiveness have been found to be able to induce moral elevation (e.g., Aquino et al., 2011). We contrasted the elevation versus amusement condition as done in previous research (e.g., Schnall et al., 2010; Schnall and Roper, 2012) to control for general positive affect. The reason behind this procedure is that positive affect alone can be a driving condition for a broader scope of attention (Rowe et al., 2007) and for a broader thought-action-repertoire (Fredrickson, 2001). Since elevation itself resembles a positive emotion, including a positive affect condition (like e.g., amusement or mirth), it was crucial to show the sole effects of elevation on behavior over and above positive affect (for evidence that elevation entails positive affect, yet cannot be reduced to it, see e.g., Strohminger et al., 2011).

Following the viewing of the respective video the participants completed a manipulation check (to examine the feelings of elevation or amusement between the conditions) and afterward three tasks: A dictator game, an ultimatum game, and responding to a request for a volunteering opportunity (the three tasks were counterbalanced to avoid sequencing effects). The exact instructions can be found in the Supplementary Material to this article (Supplementary Appendix B).

In the dictator game participants were assigned the role of the allocator. In our version of the game the allocator gets to unilaterally decide how to split a hypothetical $100 between themselves and an unknown recipient (cf. Hilbig et al., 2013). Hence, higher allocations in the dictator game can be regarded as an indicator of higher active cooperation, that is, the allocator has the choice of non-exploitation of the other, despite the allocator having the power to exploit the other without facing retaliation.

In the ultimatum game participants are responders. Our version of the game plays in a university classroom setting. The proposer is another student that has been given 100 bonus points of course credit by the professor and can offer any amount of it to the responder. If the responder accepts the offer, then it is split as proposed by the proposer. But if the responder rejects the offer, than the whole 100 course bonus points are lost to both of them. Similar to the variation used by Hilbig et al. (2013), the participant is asked to make a decision about what is the least amount they would accept without rejecting the offer. From a justice point of view, many responders will reject anything less than 50%; but from a compassion point of view, accepting any offer is viable. Therefore, low accepted offers within the ultimatum game can be regarded as an indicator of higher reactive cooperation, as it gives the participants an opportunity to offer non-retaliation, even in the face of some exploitation by another person. In both the dictator and ultimatum game only one round was played.

The volunteering opportunity consisted of a single question: “We have another research project we are currently conducting. We are having trouble finding students to participate, because participating is time-consuming and difficult, and we cannot offer any points or compensation for participation. Would you be willing to let us contact you to participate in this other study?” Response options were: “No” and “Yes” (in half of the cases “Yes” is offered before “No” to counterbalance sequence effects). Volunteering opportunities like these have been used in prior studies to measure prosocial or altruistic action tendencies (e.g., Schnall et al., 2010).

### Measures

Currently, there is no standard measure available for elevation as a state (Thomson and Siegel, 2013; Pohling and Diessner, 2016). Therefore, we selected items frequently used across studies [all items were taken from Schnall et al. (2010) for other studies using the same items, see e.g., Diessner et al., 2013; Thomson and Siegel, 2013]. In the present study, we focused on affective reactions, physical sensations, and motivational tendencies as target components of elevation (cf. Pohling and Diessner, 2016). We constructed a two item rating scale to measure *elevating emotions* (“morally uplifting,” “warmth in chest”) and another two item scale for assessing the *desire to be a better person* (“I wanted to help others,” “I wanted to become a better person”). Further, we used three items (“funny,” “laughing,” “amusing”) to assess the level of amusement or joy. As a manipulation check participants were asked to report how they felt immediately after watching the respective video clip, using a 5-point Likert-type scale from 1 (*not at all*) to 5 (*very much*).

An exploratory factor analysis (principal axis factoring) followed by a direct oblimin rotation (δ = 0) was used to examine the factor structure underlying the responses of the participants to the emotion items. Scree plot-analysis and Kaiser-criterion revealed a two factor solution – the first factor for all elevation items (rotated item loadings ranged from 0.72 to 0.87, coefficients were taken from the pattern matrix) and the second for amusement (rotated item loadings ranged from 0.83 to 0.92, coefficients were taken from the pattern matrix). The correlation of the rotated factors was between –0.39; together they explained 80.05% of the total variance. Based on these results, the items for each factor were averaged to form a scale. Internal consistencies were α = 0.91 for elevation (subscales: α = 0.82 for elevating emotions, α = 0.97 for desire to be better) and α = 0.91 for amusement.

The *revised Engagement with Beauty Scale* (EBS-R, Pohling et al., unpublished; for the EBS see Diessner et al., 2008) is an 18-item self-report scale indicating various levels of cognitive and emotional engagement concerning beauty and consists of four subscales, with the Engagement with Moral Beauty (EBS-R Moral) subscale being the relevant one in this study. The EBS-R Moral scale uses a 7-point Likert-type scale ranging from 1 (*very unlike me*) to 7 (*very much like me*), on questions such as, “When perceiving an act of moral beauty I find that I desire to become a better person.” Previous studies have shown the EBS-R Moral scale to be effective in predicting levels of moral elevation and to have high internal consistency (Diessner et al., 2013); in our current study it demonstrated an α of 0.92.

The *HEXACO-PI-R 100* scale assesses the HEXACO personality framework (Lee and Ashton, 2004) which consists of six factors: Honesty-Humility, Emotionality, Extraversion, Agreeableness (vs. Anger), Conscientiousness, and Openness to Experience. In the present study, we focused on Honesty-Humility with its facets Fairness, Sincerity, Modesty, and Greed Avoidance, and on Agreeableness (vs. Anger) with its facets Forgivingness, Gentleness, Flexibility, and Patience. Each factor comprises 16 items (4 items for each facet). The anchors range, on a 5-point Likert-type scale, from 1 (*strongly disagree*) to 5 (*strongly agree*). Alpha reliabilities in our study were α = 0.78 for H-H and α = 0.81 for AG [facets between α = 0.60 (Gentleness) and α = 0.80 (Patience)], which are comparable to previous studies (Lee and Ashton, 2004).

To assess Need for Cognition, the 18-item *NFC short scale* by Cacioppo et al. (1984) was used. Each item included a statement scored on a 5-point Likert scale ranging from 1 (*strongly disagree*) to 5 (*strongly agree*). Examples of items used in the questionnaire are: “I would prefer complex to simple problems” or “I only think as hard as I have to” (reverse scored). Cronbach’s alpha was 0.84 which is comparable to Cacioppo et al. (1984).

### Statistical Analysis

All data files, statistical analyses (syntax files), and SPSS output files are freely available at OSF (Pohling et al., 2019). At first, we analyzed the main effects of the elevating video condition versus the control condition on subsequent prosocial behavior using difference tests (*t*-test, *F*-test, Fisher’s exact test). We then investigated the interaction effects of the personality traits and video-condition on the state of elevation ratings and subsequent prosocial intention and behavior (dictator game, ultimatum game, volunteering) within a simple moderation model using the SPSS macro-script PROCESS (model 1 of PROCESS release 2.16; Hayes, 2013). We performed one moderation model separately for each personality variable as a potential moderator. In these analyses we further controlled for age and gender since both variables have been repeatedly found to be related to moral functioning in general and moral elevation in particular (cf. Pohling and Diessner, 2016; cf. Aquino et al., 2011). This finding was replicated in the present study: women and younger participants were more susceptible to elevation (Supplementary Appendix A – Figures A1–A3).

For the visual presentation of the moderating effects we used the Johnson-Neyman technique and marginal-effects plots in conjunction with visual depictions of simple slope using small multiples (all these visualization were created with the R-based tool interActive, McCabe et al., 2018). Throughout the whole result section, we first report the results calculated with the trimmed sample and second we report the results calculated with the untrimmed sample (for the composition of these samples, see above).

Note that we did not mean center the variables for conducting the moderation analysis since, although sometimes conducted for various reasons, mean centering is not required to perform a valid moderation analysis (cf. Hayes, 2013). Further, we report unstandardized regression weights. In terms of missing values, we used pairwise deletion for reporting the correlation analysis and we used listwise deletion for all other analyses.

## Results

### Descriptive Statistics and Manipulation Check

Quantile-quantile-plots and parameters for skewness and kurtosis revealed that all scales showed approximately normal distributions, except for the dictator game (trimmed sample: skewness = 0.15; kurtosis = 0.68; untrimmed sample: skewness = 0.55, kurtosis = 0.18) and the ultimatum game (trimmed sample: skewness = 0.70; kurtosis = –0.06; untrimmed sample: skewness = 0.94, kurtosis = 0.37). We then performed an outlier analysis as preparation to conducting multiple linear regression analysis. According to Field (2013), we used *z*-scores to analyze which cases can be regarded as extreme score (*z*-score > 3.29), potential outlier (*z*-score > 2.58), or probable outlier (*z*-score > 1.96). We calculated *z*-scores for all scales we investigated within our study. However, again following the recommendations of Field (2013), we only regarded extreme scores as actual outlier, since those values are statistically not likely to occur and thus can be regarded as outliers – a value that is likely not part of the target population (see Field, 2013). Following this procedure, we identified one case with an extreme score on H-H and, thus, removed it from our sample which resulted in *N* = 118 for the trimmed sample. Table 1 shows descriptive statistics and correlations for the present study using the trimmed sample.

**FIGURE 1:**
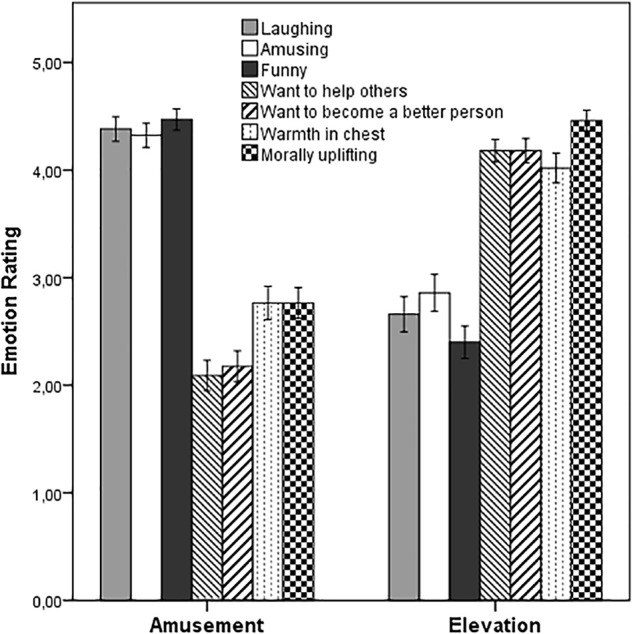
Mean self-reported feelings and appraisals from participants as a function of video condition. Error bars indicate standard errors. The results depicted are those using the trimmed sample, *N* = 118.

The emotion-induction was successful: Participants reported high levels of elevation only in the elevation condition and high levels of amusement only in the control condition (see Figure 1). A MANOVA using the elevation scale and the amusement scale as dependent variables confirmed a main effect of video-condition, Pillai’s trace: *V* = 0.74, *F*(2, 115) = 159.80, *p* < 0.001, η^2^ = 0.74, trimmed sample. Separate univariate ANOVAs on each emotion scale revealed significant effects of state elevation, *F*_Welch_ (1, 115.75) = 151.44, *p* < 0.001, η^2^ = 0.54, and state amusement, *F*(1, 117) = 118.47, *p* < 0.001, η^2^ = 0.51 (Bonferroni level: *p* = 0.025). These results could be replicated within the untrimmed sample: Pillai’s trace was *V* = 0.75, *F*(2, 141) = 206.29, *p* < 0.001, η^2^ = 0.75. Separate univariate ANOVAs on each emotion scale again revealed significant effects of state elevation, *F*_Welch_(1, 137.76) = 211.94, *p* < 0.001, η^2^ = 0.59, and state amusement, *F*(1, 143) = 106.72, *p* < 0.001, η^2^ = 0.43 (Bonferroni level: *p* = 0.025).

### Main Effect of Elevation on Prosocial Intentions and Behavior (H1)

**FIGURE 2:**
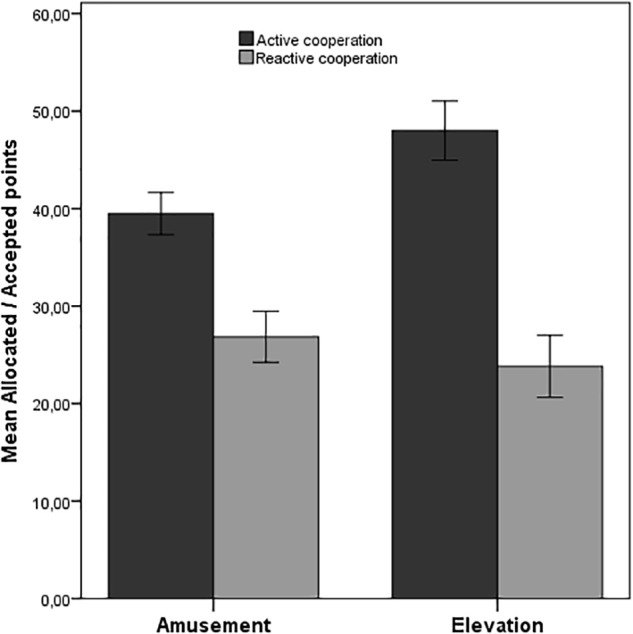
Mean amounts of allocated points in the dictator game (active cooperation) and mean minimal accepted points in the ultimatum game (reactive cooperation). Error bars indicate standard errors. The results depicted are those using the trimmed sample; for dictator game: *N* = 116, for ultimatum game: *N* = 117.

On average, participants in the elevation condition shared significantly more money in the dictator game (*M* = 48.00, *SD* = 20.98) than did those in the control condition (*M* = 39.21, *SD* = 17.66), *t*(114) = –2.44, *p* < 0.05, two-tailed, *d* = 0.45, trimmed sample (see Figure 2). The same effect was found using the untrimmed sample: dictator game (*M* = 57.06, *SD* = 26.86) vs. control condition (*M* = 41.46, *SD* = 21.89), *t*(140) = –3.32, *p* < 0.001, two-tailed, *d* = 0.64. These findings provided strong evidence for H1a.

Further, in line with H1b, participants in the elevation condition accepted lower offers in the ultimatum game (*M* = 23.18, *SD* = 21.86) than did those in the control condition (*M* = 26.85, *SD* = 21.51), however, this difference was not significant, *t*(115) = 0.91 *p* = 0.37, two-tailed, *d* = 0.17, trimmed sample (Figure 2). This effect was also not significant within the untrimmed sample: elevation (*M* = 21.75, *SD* = 24.46) vs. control condition (*M* = 23.96, *SD* = 21.80), *t*(141) = 0.57, *p* = 0.57, two-tailed, *d* = 0.10.

With regard to volunteering, the elevation condition (*n* = 17 “yes,” *n* = 32 “no”) did not differ from the control condition (*n* = 30 “yes,” *n* = 37 “no”), χ^2^ = 1.19, Fisher’s exact test, *p* = 0.34, two-tailed, trimmed sample. The same data pattern was found using the untrimmed sample: the elevation condition (*n* = 27 “yes,” *n* = 39 “no”) did not differ from the control condition (*n* = 34 “yes,” *n* = 41 “no”), χ^2^ = 0.28, Fisher’s exact test, *p* = 0.61, two-tailed.

### Personality and Susceptibility to Elevation (H2)

In contrast to H2, none of the investigated traits (EmB, H-H, AG, and NFC) moderated the susceptibility to elevation (see Table 2). For exploratory reasons, we analyzed the complete elevation scale as well as the subscales.

**Table 2:** Moderation analyses.

	Moral elevation^1^		Prosocial behavior^1^		Prosocial intention^2^	
	Elevation ratings after watching elevating video clip		Dictator game generosity (active cooperation)		Ultimatum game acceptance rate (reactive cooperation)		Willingness to volunteer	
Interaction term	*B*	*t*	*B*	*t*	*B*	*t*	*B*	Z
Video × H-H	-0.15	-0.43	-9.49	-1.18	8.46	0.93	1.34	1.30
Video × AG	0.08	0.27	3.27	0.46	-3.55	-0.45	1.22	1.39
Video × EmB	0.05	0.39	**7**.**53**	**2**.**36**^∗^	-3.11	-0.87	0.39	0.86
Video × NFC	0.07	0.26	**20**.**99**	**3**.**21**^∗∗^	-5.70	-0.76	0.94	0.91

*H-H = Honesty-Humility, AG = Agreeableness vs. Anger, EmB = Engagement with Moral Beauty, NFC = Need for Cognition, dictator game: higher values reflect higher active cooperation; ultimatum game: lower values reflect higher reactive cooperation; volunteering: 1 = no, 2 = yes, Video: 1 = amusement condition, 2 = elevation condition. Control variables included in the regression model: age, gender. The regression weights depicted are those using the trimmed sample. Due to missing values sample sizes varied between 85 < N < 86. ^1^Multiple linear regression, ^2^logistic regression. ^∗∗∗^p < 0.001, ^∗∗^p < 0.01, ^∗^p < 0.05, ^†^p < 0.10, all tests two-tailed; all ps < 0.05 in bold.*

### Personality and Behavioral Effects of Elevation (H3)

**FIGURE 3:**
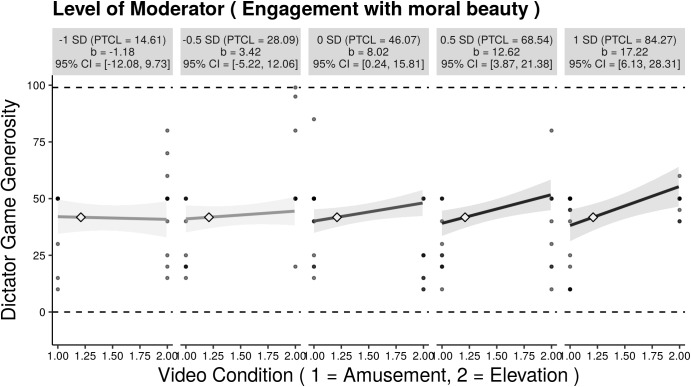
Small-multiples depictions of the interaction effect of video condition and Engagement with moral beauty (the moderator) on dictator game generosity (active cooperation). The small multiples illustrate the interaction across the range from 1 SD below to 1 SD above the mean of Engagement with moral beauty. Each graphic shows the computed 95% confidence region (shaded area), the observed data (gray circles), the maximum and minimum values of the outcome (dashed horizontal lines), and the crossover point (diamond). CI = confidence interval; PTCL = percentile. The results depicted are those using the trimmed sample, *N* = 85.

**FIGURE 4:**
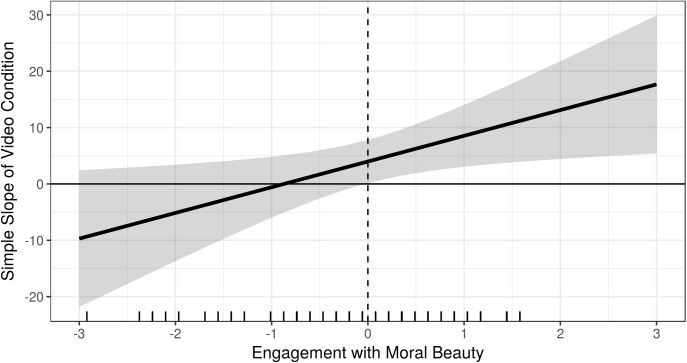
Marginal effects (or regions-of-significance) plot: On the *y*-axis we depicted the simple slope of video condition on dictator game generosity. On the *x*-axis we *z*-transformed Engagement with moral beauty for the purpose of this figure. The simple slope is significant and positive when Engagement with moral beauty is above-average. 40.68% of observations in Engagement with moral beauty are within this region. The results depicted are those using the trimmed sample, *N* = 85.

**FIGURE 5:**
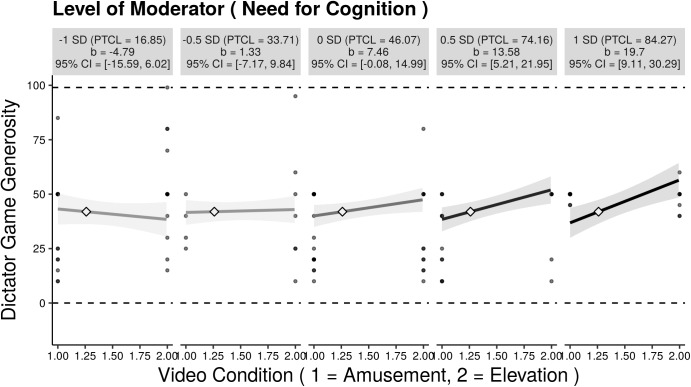
Small-multiples depictions of the interaction effect of video condition and Need for Cognition (the moderator) on dictator game generosity (active cooperation). The small multiples illustrate the interaction across the range from 1 SD below to 1 SD above the mean of Need for Cognition. Each graphic shows the computed 95% confidence region (shaded area), the observed data (gray circles), the maximum and minimum values of the outcome (dashed horizontal lines), and the crossover point (diamond). CI = confidence interval; PTCL = percentile. The results depicted are those using the trimmed sample, *N* = 85.

**FIGURE 6:**
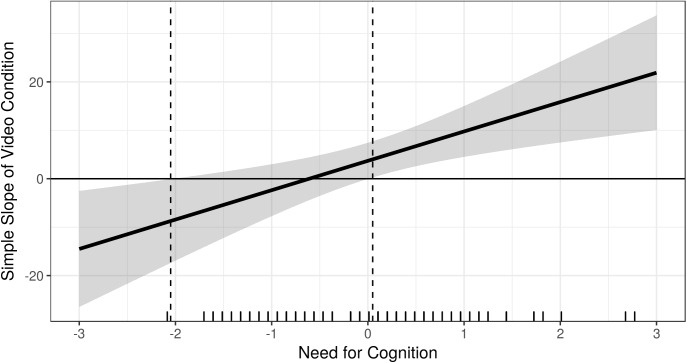
Marginal effects (or regions-of-significance) plot: On the *y*-axis we depicted the simple slope of video condition on dictator game generosity. On the *x*-axis we *z*-transformed Need for Cognition for the purpose of this figure. The simple slope is significant and negative when NFC is –2.05 standard deviations away from the mean or further. 0.85% of observations in NFC are within this region. The simple slope of the elevating video condition on dictator game generosity is significant and positive when NFC is 0.05 standard deviations away from the mean or further. 38.98% of observations in NFC are within this region. The results depicted are those using the trimmed sample, *N* = 85.

In line with H3a and H3d, the interaction terms between active cooperation and EmB (trimmed sample: *b* = 7.53, *t*(79) = 2.36, *p* < 0.05) and NFC (trimmed sample: *b* = 20.99, *t*(79) = 3.21, *p* < 0.01), respectively, were found to be significant (see Table 2). Higher scores in EmB were associated with allocating more money in the elevation condition in contrast to participants low on EmB (see Figure 3). That is, the higher the EmB the stronger the effect of video-induced elevation on dictator game generosity (Figure 4). Likewise, higher scores in NFC were associated with allocating more money in the elevation condition in contrast to participants low on NFC (see Figure 5). That is, the higher the NFC the stronger the effect of video-induced elevation on dictator game generosity (Figure 6). These effects could be replicated using the untrimmed sample; however, the interaction between video condition and NFC was not significant anymore: EmB: *b* = 11.19, *t*(102) = 3.28, *p* < 0.01; NFC: *b* = 14.82, *t*(102) = 1.90, *p* = 0.06. All these effects were restricted solely to active cooperation (dictator game generosity), no significant effects could be found for ultimatum game behavior and the willingness to volunteer (see Table 2).

In contrast to our hypotheses, no interactions were found between video-condition and H-H or AG (H3b and H3c) for any of the behavioral measures or prosocial intentions (Table 2). However, we replicated the correlation between H-H and active cooperation (dictator game generosity, *r* = 0.31, *p* < 0.01), as found by Hilbig et al. (2013), but we could not replicate the correlation between AG and reactive cooperation (ultimatum game behavior, *r* = –0.07, *p* = 0.53). Rather, in contrast to Hilbig et al. (2013), AG was also positively related to active cooperation (*r* = 0.22, *p* < 0.05) so that no double dissociation between H-H, AG dictator game, and ultimatum game behavior was found. Furthermore, we found positive correlations between EmB and volunteering (*r* = 0.22, *p* < 0.05) and between NFC and volunteering (*r* = 0.36, *p* < 0.001). Lastly, we found no correlation between NFC and ultimatum game behavior.

## Discussion

The present experiment is among the few demonstrating the effects of moral elevation on behavior in economic games (Zhao and Smillie, 2015). We found that elevation fostered active cooperation (reflected by higher allocations of money in a dictator game). Moreover, we found that EmB and NFC moderated the effects of elevation on active cooperation. These results replicate and extend empirical findings showing that experimentally induced moral elevation prompts subsequent prosocial action (for a summary of prior studies, see Pohling and Diessner, 2016; Thomson and Siegel, 2017). However, we found no effects of elevation on reactive cooperation (reflected by lower ultimatum game acceptance rates) or prosocial intentions (reflected by the intention to volunteer in an additional time-consuming task). Taken together, elevation promoted only one of our three dependent prosocial variables, namely the one that was most closely related to altruism: dictator game generosity (Baumert et al., 2014).

Referring to the main effect of elevation, our correlation analysis exemplified that the effects of the elevating video condition on active cooperation were indeed caused by higher levels of state elevation and not just by lower levels of amusement: only the state elevation but not the amusement ratings correlated with dictator game generosity. Since dictator game generosity can be regarded as an expression of altruism (e.g., Baumert et al., 2014; Zhao and Smillie, 2015; Thielmann and Hilbig, 2018), our findings corroborate prior research on the link between elevation and altruistic or prosocial behavior (cf. Pohling and Diessner, 2016).

Most importantly, the current study aimed at investigating the moderating role of personality traits on the susceptibility for moral elevation and its behavioral effects. We found that NFC and EmB promoted the behavioral effects of experimentally induced moral elevation. That is, higher scores on these traits were a condition for higher levels of active cooperation within the elevation condition. Our findings are in line with Thomson and Siegel (2013), insofar that certain conditions – in our case specific personality traits – maximize the behavioral effects of elevation but not the level of reported elevation itself. They found the same effect for the manipulation of the elevating story (the perceived effort to perform the moral deed or the character of the recipient of the moral act). Perhaps EmB and NFC are traits that foster the perception of these aspects of a moral situation that elicited elevation (effort and character of the recipient). NFC might do this by means of improving the cognitive understanding of what it actually takes to perform a specific moral act or whether the character of the recipient of the moral act is good or bad or whether the moral act in question indeed does lead to good consequences (for empirical evidence that NFC is linked to cognitive empathy/perspective taking, see Strobel et al., 2017). In doing so, NFC might foster the translation of elevation into action.

A similar effect might be true for EmB. However, we assume that EmB, through its close relation to self-transcendent emotions and affective empathy (Diessner et al., 2008; Smith et al., 2011; Pohling, 2018), is likely to broaden the scope of attention as it was proposed and found by research on the broaden-and-built theory of positive emotion (cf. Fredrickson, 2001) and, thus, help to see more details about the moral act in question and its consequences. As result, higher levels of EmB might also facilitate the evaluation of the character of a moral story and, in consequence, promote the behavioral effects of moral elevation. Yet, the concrete mechanism behind the moderation effects of EmB and NFC found in this study needs further empirical clarification.

Our findings also corroborate Diessner et al. (2013) since we found evidence for the moderating role of EmB in moral elevation. However, in our study only the behavioral effects of elevation were moderated and not the level of state elevation ratings, as was the case in Diessner et al. (2013). Rather, our study found a direct association between EmB and prosocial intentions (volunteering). That is, individuals who score high on trait elevation tend to respond in a prosocial way independent of the respective situation, which replicates former studies on the relationship of trait elevation and prosocial intentions and behavior (cf. Pohling and Diessner, 2016).

In contrast to Diessner et al. (2013), we used another video stimulus to induce elevation. Whereas Diessner et al. (2013) used a video that depicted a sport event where the members of the opposing team helped an injured baseball player to make her home run, we used a Thai commercial video that vividly showed a moral agent performing several consecutive examples of helping behavior, in particular caring for others in need, without getting a direct reward for it. Watching caring moral exemplars has been considered the most potent elicitor for moral elevation (cf. Oliver et al., 2012). However, which aspects of a moral act (or which kind of virtuous behavior at all), have the power to elicit elevation is still an open empirical question (e.g., Oliver et al., 2012; Pohling and Diessner, 2016). Therefore it is difficult to say what caused the differences between our study and the study by Diessner et al. (2013) but the video material could be one possible explanatory factor that needs further investigation.

Interestingly, we found no moderating effects on the dependent variables of reactive cooperation (ultimatum game behavior) and prosocial intentions (volunteering). In the case of reactive cooperation, a possible explanation for our results could be the university context that was part of our version of the ultimatum game. In our version of the game, allying against the professor, so that she does not take away the course credits, might be a possible alternative motivation that perhaps weakened the effects of the experimental manipulation or the effects of any of the moderators. Put differently, in the competitive setting of the university, accepting any offer, even when it is very low, might be the most prudent option for the responder in the game. Recent studies have found that individuals with higher levels of NFC tend to accept more fair offers and reject more unfair offers within the ultimatum game paradigm (Mussel et al., 2014) reflecting a more deliberate and rational thinking style. In contrast, we found no association between NFC and ultimatum game behavior. However, the aforementioned study of Mussel et al. (2014) used another methodology than the present one: participants played a series of 46 short trials and the game was not embedded into a specific context as was ours. These differences could be a methodological explanation for the different results of our and the study of Mussel et al. (2014).

Alternatively, the non-significant findings with regard to ultimatum game behavior could be due to the nature of the measure in general. Since ultimatum game responder behavior entails the power to retaliate for unfair offers, fairness concerns play a greater role than in the dictator game (Baumert et al., 2014). Thus, ultimatum game rejection decisions can be seen as a blend of a measure of prosocial behavior and strategic fairness (cf. Baumert et al., 2014). Baumert et al. (2014) found that ultimatum game rejection decisions correlated with competitive social value orientation and a need for power, with low to moderate effect sizes. They therefore concluded: “It appears that this kind of behavior might be motivated by a concern to avoid one’s own relative disadvantages in comparison to others” (Baumert et al., 2014, p. 189). Perhaps this self-focused motivation that is associated with ultimatum game rejections decisions might be one reason why elevation fostered such kind of fairness-related behavior not so strongly. By the way, the conceptual difference of our three dependent variables is also reflected by the non-correlation between all of them (see Table 1).

Furthermore, we found no moderating, but only direct effects between prosocial intentions (volunteering) on the one hand and higher levels of NFC on the other hand. This moderate association of NFC and prosocial intentions might be another indicator that NFC is a moral capacity which possibly fosters not only moral thoughts but also moral intentions (see e.g., Strobel et al., 2017). However, this finding could also be interpreted without any moral implications. Individuals with higher levels in NFC might simply prefer the additional time-consuming task since it resembles a possibility to learn new things which is in line with the construct of NFC (e.g., Cacioppo and Petty, 1982). Thus, the mechanisms behind the association of NFC and volunteering need further empirical clarification.

Finally, in contrast to our hypotheses, we did not find any moderating effect for AG and H-H. Thus, both traits do not seem to be a condition for the susceptibility to experience moral elevation or its behavioral effects. Rather H-H as well as AG correlated positively with active cooperation. Thus, although it was not the explicit aim of this study, we could not replicate the double dissociation between active/reactive cooperation and H-H/AG that was found by Hilbig et al. (2013). Again, this finding might be due to slight methodological differences in our ultimatum game that were already discussed above.

Some potential limitations and future directions should be mentioned: Hilbig et al. (2013) summarized that economic games might not be pure measures of active or reactive cooperation, respectively. There is a variety of factors influencing the behavior in those games, such as short-term situational factors, social norms, or expectations of the behavior of others (cf. Hilbig et al., 2013). Further, we theoretically implied that volunteering in an additional time-consuming task within the university context can be regarded as prosocial or altruistic intention; however, whether or not the actual motivation driving this choice is indeed prosocial or altruistic (cf. Batson, 2010) wasn’t measured in this study. According to Clary and Snyder (1999) there are several motives to volunteer ranging from humanitarian/prosocial to more self-focused motivations, such as reducing guilt or just gaining new experiences. This variety of motivations to volunteer might explain why we found almost no effects for this dependent variable. Future research should ensure that the motivation behind volunteering is indeed prosocial or altruistic. Another promising approach for future research on the link between elevation and volunteering might be the volunteer’s dilemma (Krueger, 2019). In a volunteer dilemma a prosocial act leaves the volunteer better off than if no one had volunteered but worse off than if someone else had volunteered. We therefore think the volunteering paradigm could be fruitful for elevation research, (1) to investigate the effects of elevation on volunteering in greater detail, (2) to investigate the motives underlying volunteering that it elicited by elevation, and (3) whether elevation indeed induces volunteering behavior that includes a real sacrifice which was proposed by prior research (Haidt, 2003a).

To control for experimenter demand effects we deleted those participants who reported to allocate all their money within the dictator game or accepted even zero offers with the ultimatum game since such outcomes typically do not occur in bargaining games (see above). However, the most elegant way to deal with the problem of experimenter demand effects (e.g., Bekkers, 2007; Moshagen et al., 2011) within bargaining games would have been a so called double blind dictator game (Hilbig et al., 2015) in which the choice is not visible for the experimenter and the receiver of the money is also unknown for the dictator. At last, the sample size was relatively small, especially after trimming the extreme responses in the economic games. Despite that, we could replicate most of our findings when we used the untrimmed sample.

## Conclusion

At the beginning of this article we asked who is not moved and inspired by acts of moral beauty like those Oskar Schindler showed. The results of the present experiment indicate that individual differences on NFC and EmB are crucial when it comes to translating moral elevation into active prosocial behavior. People who show higher levels of NFC and EmB are more easily inspired to do good things themselves when they experience the moral emotion of elevation in contrast to people with lower expressions of these traits. These results not only inform future experimental research on elevation to include these traits as moderating variables but they also exemplify the individual boundaries of large-scale moral elevation interventions.

## Ethics Statement

This study was carried out in accordance with the recommendations of the American Psychological Association. All subjects gave written informed consent in accordance with the Declaration of Helsinki. The protocol was approved by the Institutional Review Board of the Lewis-Clark State College, ID, United States (IRB Proposal FS-14-03).

## Author Contributions

RP and RD planned the study. RD, SS, and DW gathered the data. RP statistically analyzed the data. RP, RD, and AS wrote the manuscript.

## Conflict of Interest Statement

The authors declare that the research was conducted in the absence of any commercial or financial relationships that could be construed as a potential conflict of interest. The handling Editor declared a past co-authorship with one of the authors AS.

## Footnotes

1 Altruism can be defined as a motivational state with the ultimate goal of increasing other’s welfare (Batson, 2010).

2 https://www.youtube.com/watch?v=cZGghmwUcbQ

3 https://www.youtube.com/watch?v=qy_mIEnnlF4
