# Supplementary material for: Moral Elevation and Economic Games: The Moderating Role of Personality
Source: Front Psychol. 2019 Jun 14;10:1381. doi: 10.3389/fpsyg.2019.01381 (PMC6587376; doi:10.3389/fpsyg.2019.01381)
Supplement: Supplementary file 1 [file Table_1.docx]

**Appendix A. Supplementary analysis**

To explore the role of age and gender as potential moderators of experimentally-induced state elevation, we performed a further moderation analysis. In line with previous studies (for an overview see [Pohling & Diessner, 2016](#_ENREF_1); [Thomson & Siegel, 2017](#_ENREF_2)), a simple moderation model with the experimental condition as independent variable and the state elevation scale as dependent variable showed that women were more easily moved by the elevating video clip then men, however, this interaction was only significant within the bigger / untrimmed sample (trimmed: *b* = .-51, *t*(83) = -1.57, *p* = .12; untrimmed sample: *b* = .-69, *t*(106) = -2.37, *p*  < .05). That is, women reported higher state elevation ratings in response to watching the uplifting video clip then men (figure A1). Second, younger participants were more easily elevated than older participants, but here the moderation was only found within the trimmed sample: *b* = -.13, *t*(82) = -2.38, *p* < 0.05; untrimmed sample: *b* = -.04, *t*(105) = -1.07, *p* = 0.28 (see figure A2-A3). In the smaller / trimmed sample, age ranged from 18 to 32 years, whereas in the bigger / untrimmed sample, it ranged from 18 to 50 years (with four participants being older than 32). That is, age showed a significant moderation only when the age range was restricted. This could be an indication that either this finding is an artifact of the smaller sample or the moderating role of age is perhaps restricted to a specific age-range.


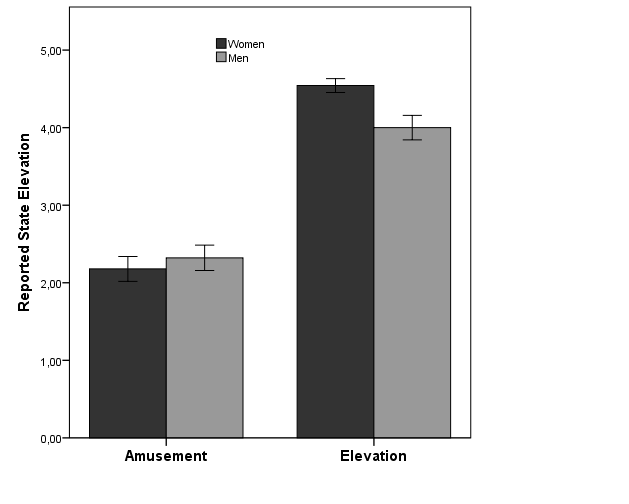


*Figure A1.* Interaction effect of video condition and gender (the moderator) on reported state elevation after watching the elevating video clip. Error bars indicate standard errors. The results depicted are those using the untrimmed sample, *N* = 110.


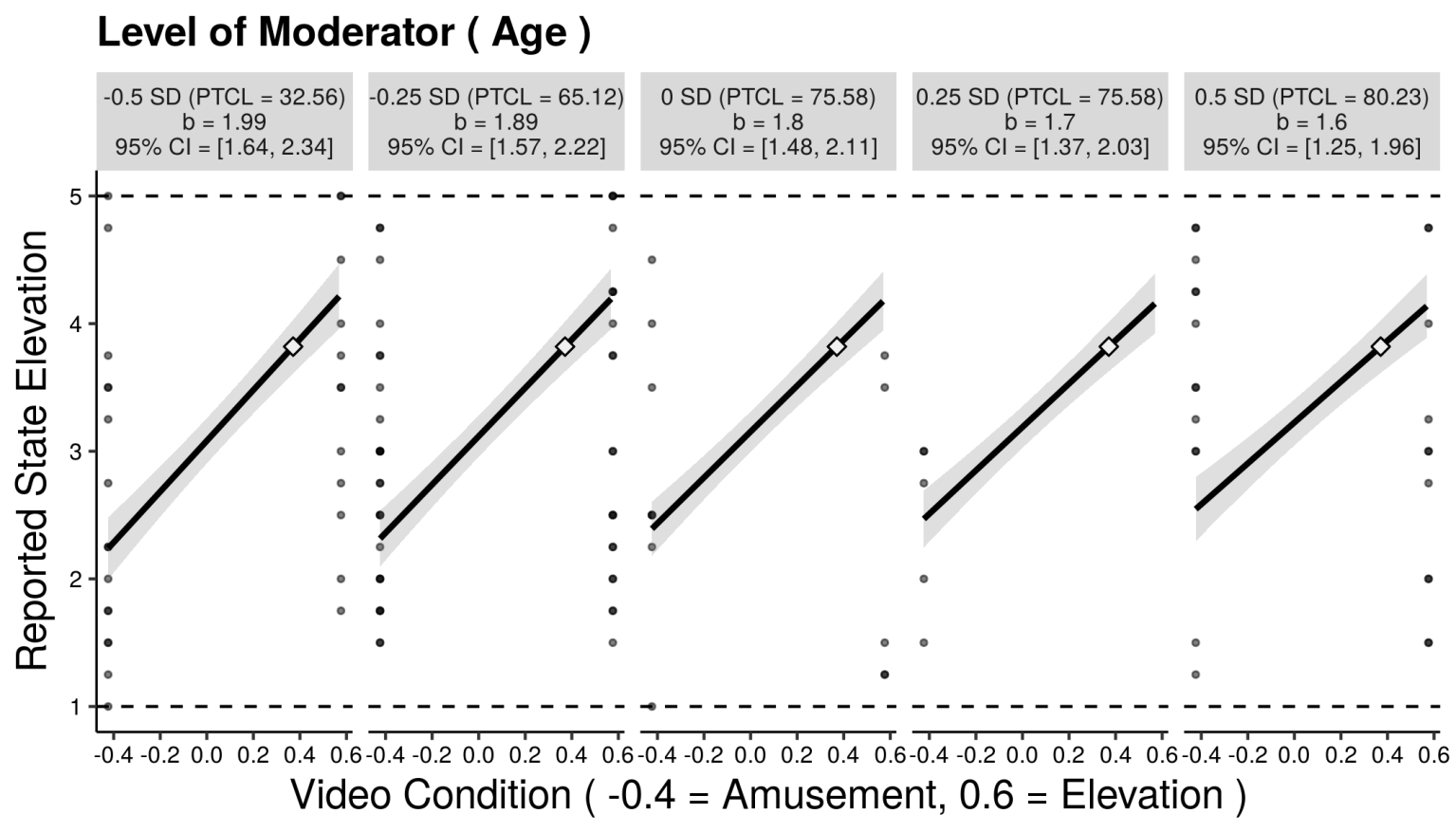


*Figure A2.* Small-multiples depictions of the interaction effect of video condition and age (the moderator) on reported state elevation after watching the elevating video clip. The small multiples illustrate the interaction across the range from 0.5 SD (1.5 years) below to 0.5 SD (1.5 years) above the mean of age (*M* = 20.8 years). Each graphic shows the computed 95% confidence region (shaded area), the observed data (gray circles), the maximum and minimum values of the outcome (dashed horizontal lines), and the crossover point (diamond). CI = confidence interval; PTCL = percentile. To improve the visualization of these plots, we had to mean center the independent as well as the moderator variable. The results depicted are those using the trimmed sample, *N* = 86.

*

*

*Figure A3.* Marginal effects (or regions-of-significance) plot: On the y-axis we depicted the simple slope of video condition on reported state elevation. On the x-axis we z-transformed age for the purpose of this figure. The simple slope is significant and positive when age ranges from -0.7 SD (18.0 years) to 2.4 SD (27.4 years). 69.49% of observations in age are within this region. The results depicted are those using the trimmed sample, *N* = 86.

**Appendix B. Supplementary materials**

**Manipulation Check:**

In regard to the short video that you just watched, please answer the following on this 5-point scale:

1 Not at all 2 Some 3 Medium 4 Much 5 Very much

Please circle the appropriate number.

After watching that video I felt:

1. …I wanted to help others: 1 2 3 4 5

2. …I wanted to become a better person: 1 2 3 4 5

3. …it was funny: 1 2 3 4 5

4. …warmth in chest: 1 2 3 4 5

5. …like laughing: 1 2 3 4 5

6. …it was morally uplifting: 1 2 3 4 5

7. …it was amusing: 1 2 3 4 5

**Dictator Game (Active Cooperation):**

“A. Division of $100

You are given $100 and you are asked to divide them between yourself and a complete stranger that you will never meet, is anonymous, and will never know who you are.

Your task: How many dollars (of the available $100) do you give to this other anonymous stranger?

Out of the $100, I allocate ________ to the other. I keep the rest.”

**Ultimatum Game (Reactive Cooperation):**

“B. Accepting or Rejecting points

This is a completely different situation and is not related to the Division of 100 points mentioned above.

In this situation a student in class is given 100 bonus points by the professor and asked to share them with you. This other student can offer you any amount of the 100 points. However, if you do not like their offer, you can reject it. But, if you reject it, the professor takes away the 100 points, and both of you get no points. What is the minimum amount of points you would accept, without rejecting the offer?

Minimum amount of points you would accept without rejecting the offer:________________________”

**Volunteering Question (Prosocial Intention):**

“C. Question:

We have another research project we are currently conducting. We are having trouble finding students to participate, because participating is time-consuming and difficult, and we cannot offer any points or compensation for participation. Would you be willing to let us contact you to participate in this other study? Yes / No”
